# On the meaning of “plasticity” in neuroscience and mental health research and its relation to the action of psychedelic therapy

**DOI:** 10.3389/fnins.2026.1875339

**Published:** 2026-09-01

**Authors:** R. L. Carhart-Harris, R. J. Zeifman, L. Pasquini, B. Nandi, C. Rohani-Shukla, S. Suseelan, P. Mediano, F. Rosas, C. Timmermann, H. Kettner, P. A. McConnell

**Affiliations:** 1 Carhart-Harris Lab, Departments of Neurology and Psychiatry, Weill Institute for Neurosciences, University of California, San Francisco, San Francisco, CA, United States; 2 Centre for Psychedelic Research, Imperial College London, London, United Kingdom; 3 Department of Psychology, The New School for Social Research, New York, NY, United States; 4 Centre for Psychedelic Medicine, NYU Grossman School of Medicine, New YorkNY, United States; 5 Department of Neurology, Neuroscape, Weill Institute for Neurosciences, University of California, San Francisco, San Francisco, CA, United States; 6 Department of Neurology, Carhart-Harris Lab, Weill Institute for Neurosciences, University of California, San Francisco, San Francisco, CA, United States; 7 Department of Experimental Psychology, Psychology and Language Sciences, University College London, London, United Kingdom; 8 Centre for Neuroimaging Sciences, King’s College London, London, United Kingdom; 9 Department of Computing, Imperial College London, London, United Kingdom; 10 School of Engineering and Informatics, University of Sussex, Brighton, United Kingdom; 11 Centre for Eudaimonia and Human Flourishing, University of Oxford, Oxford, United Kingdom; 12 Centre for Consciousness Research, University College London, London, United Kingdom; 13 Department of Psychiatry and Behavioral Sciences, Carhart-Harris Lab, Weill Institute for Neurosciences, University of California, San Francisco, San Francisco, CA, United States

**Keywords:** allostatic recalibration, critical-period reopening, entropy of spontaneous brain activity, Hebbian plasticity, Lempel-Ziv complexity, maladaptive plasticity, psilocybin, psychological flexibility

## Abstract

First-line dictionary definitions of “plasticity” describe it as the ability (of a thing) to be shaped or molded; we call this “plasticity proper” (PP). In neuroscience, however, “neural plasticity” or “neuroplasticity” is defined as an induced change in brain function or structure. These two plasticities are not the same. Several popular biomarkers of neuroplasticity index part of an evoked process, often linked to learning. Paradoxically, these processes often bias phenotypic canalization, the opposite of phenotypic plasticity. Recognizing this paradox puts into question the custom of using “plasticity” as a shorthand for all neuroplastic phenomena. This custom risks an overgeneralization fallacy and the conflation of assays of evoked brain changes with plasticity proper. We also question extrapolating from any biomarker of neuroplasticity to improved mental health, whether it be aligned with plasticity proper or not. The relationship between the two is, logically, *context dependent*. As a resolution, we propose a new construct, “mediational and recalibrative plasticity” (MR-P) and show how markers of it can describe and predict examples of phenotypic plasticity, where those phenotypes are states and traits relevant to mental health. In contrast to many markers of neuroplasticity, MR-P *is* aligned with plasticity proper.

## 1 Introduction

The most basic dictionary definition of plasticity states it to be: “the quality or state of being plastic,” especially the “capacity for being molded or altered” (Merriam-Webster, 2026). In this article, we refer to this orthodox plasticity as “plasticity proper” (PP). Plasticity proper is domain-general—it is a basic computational property that can be applied to any phenomenon, whether that be a property of brain function or anatomy—in the domains of neuroscience and biology, a psychological state or trait—in the domain of psychology and psychiatry, or even the malleability of a substance, such as a metal, in the domains of physics and metallurgy.

It is common to see first-line dictionary definitions of plasticity followed by an overtly biological definition, reading something such as: “the adaptability of a phenotype (i.e., a property of an organism) to changes in its environment”. This definition is aligned with PP. The effect of increasing the plasticity/changeability of a phenomenon, for example, a psychological phenotype such as a state or trait relevant to mental health, should, by implication, render that phenotype more responsive to, and potentially adaptable to, its environment. The biology-specific definition of plasticity is sometimes referred to as “phenotypic plasticity” (Agrawal, 2001). Conveniently, it is used consistently in psychology (Belsky and Pluess, 2013). It is intentional that phenotypic plasticity, psychological plasticity, and plasticity proper share the acronym “PP” as each “plasticity” recognizes *changeability* as the most basic, qualifying property.

A major motivation of the present article is to address a popular custom within neuroscience, mental health research, and popular science to unconditionally equate (if not conflate) markers of neuroplasticity with PP—and positive mental health change. In the late 19th century, inspired by recent theories and discoveries concerning neuronal and synaptic communication, the psychologist William James, and neuroscientists, Santiago Ramón y Cajal, Eugenio Tanzi, and Ernesto Lugaro, each of them referred to the notion of brain “plasticity.” All of them, and most notably James, speculated that habit formation and learning must relate to plastic changes in the brain (Berlucchi and Buchtel, 2008).

Here we argue that these earliest known uses of “plasticity” in neuroscience set the stage for a semantic divergence from PP. The understandable logic of James and others was that a change in mind or behavior must correspond with a change in brain function or anatomy, and any such changes must require a degree of plasticity in both mind and brain. This meaning of neuroplasticity—as an experience, or stimulus-induced brain change—has remained somewhat consistent over the years (Berlucchi and Buchtel, 2008; Mateos-Aparicio and Rodríguez-Moreno, 2019). For example, neuroplasticity is commonly defined as the ability of some property of brain function or anatomy to change in response to a stimulus (Mateos-Aparicio and Rodríguez-Moreno, 2019). However, in the present study, we spotlight an issue with this meaning; namely, that it relates to a neuronal event or process that can, paradoxically, predispose the mind and behavior toward the inverse of PP, namely, “canalization”.

First introduced as a philosophical image by Henri Bergson and formalized in developmental biology by Conrad Hal Waddington (Posteraro, 2022; Waddington, 1942), *canalization* describes the process of reducing phenotypic variability (the capacity to *vary* in response to genetic events or environmental pressures), whether within an organism across its development or between generations via evolutionary mechanisms. In an evolutionary context, canalization refers to the ability of a population of organisms to produce a consistent phenotype by following what Waddington called a *chreode*: a canalized developmental path (from the Greek for “necessary” and “path”) (Waddington, 1957). A canalized phenotype remains robust even in the face of environmental or genetic pressures or perturbations. Canalized phenotypes are resistant to change and variation. Plastic phenotypes exhibit the opposite tendency; they vary easily and are sensitive to influence and change.

Several types of neuroplasticity have been named and defined, with varying degrees of specificity and granularity. The following non-exhaustive list includes overlapping constructs that should not necessarily be viewed as independent: functional plasticity, Hebbian plasticity, long-term potentiation (LTP) and long-term depression (LTD), activity-dependent plasticity (ADP), spike-timing dependent plasticity (STDP), anatomical or structural plasticity, structural remodeling, synaptic plasticity, homosynaptic and heterosynaptic plasticity, spinogenesis, dendritogenesis, neurogenesis, synaptic pruning, metaplasticity, homeostatic plasticity, and synaptic scaling.

Here we spotlight a paradox whereby many popular biomarkers of increased neuroplasticity index part of a (learning) process that favors phenotypic canalization ahead of plasticity. Many of the best-known and studied markers of neuroplasticity are also, more specifically, markers of synaptic plasticity (Citri and Malenka, 2007). Focusing on maladaptive (associative) learning and canalization, two stark examples within psychiatry include the pathogenesis of chronic pain and substance use disorders (Kalivas, 2009; McConnell et al., 2019; Flor, 2008; Kalivas and O'Brien, 2007), where pain and reward circuits become sensitized, reinforced, and ultimately *canalized* in a way that comes to define the disorders, including their stubborn resistance to treatment (Carhart-Harris et al., 2023). There are many examples—like these—of associative learning and related neuroplastic mechanisms driving the development of phenotypic canalization undergirding symptoms of mental illness.

Long-term potentiation and other related Hebbian mechanisms are likely to be involved in the acquisition and entrenchment of many psychopathological habits (Citri and Malenka, 2007; Kalivas, 2009; Carhart-Harris et al., 2023; Kalivas and O'Brien, 2007). Granted, engagement of such mechanisms will not unconditionally strengthen maladaptive phenotypes; for example, LTP (and LTD) could support the learning of new, healthier, more adaptive associations and behaviors, such as can occur with psychotherapy. Still, despite such exceptions, the paradox remains—namely, that activating synaptic and neural plasticity markers (such as LTP) is associated with mechanisms that favor the canalization of maladaptive phenotypes defining mental illnesses (Citri and Malenka, 2007; Carhart-Harris et al., 2023). If, as we argue here, and elsewhere (Carhart-Harris et al., 2023), common neuroplasticity markers can index the acquisition of maladaptive phenotypes, then markers and mechanisms of extinction learning (Bouton, 2004) or “unlearning” may be more relevant and conducive to PP (Du et al., 2023; Sottile and Vida, 2022; Catlow et al., 2013; Schindler et al., 1986) than the classical markers and mechanisms of Hebbian learning, such as LTP.

In this regard, we note Cajal’s early references to early life plasticity and regeneration; phenomena that are more naturally consistent with PP (Berlucchi and Buchtel, 2008). Also better aligned with PP is “restorative plasticity” (Stahnisch and Nitsch, 2002). Perhaps the divergence between neuroplasticity and PP that began early on within the neurosciences happened because of the appeal of measuring experimentally induced changes in brain function or anatomy, rather than maintaining a respect for the primacy of phenotypic changeability itself. Put succinctly, brain *change* was privileged over *changeability*. Another related reason for the divergence is the focus that basic and preclinical neuroscience has placed on brain, biology, and behavior—and how each relates to learning, in particular. Accordingly, scientists have tended to privilege laboratory-friendly assays of neuroplastic brain changes (such as LTP-related synaptic changes) and (animal) behavioral change over PP.

It feels important—if not essential—to understand whether any given increase in neuroplasticity, in a specific context, carries the risk of driving a paradoxical narrowing of a phenotype’s capacity for change. If such a phenomenon were to occur, we might refer to it as a “paradoxically canalizing plasticity (PCP)”. Here we highlight that Hebbian mechanisms of acquisition and reinforcement learning are likely to be operative in PCP. We also consider how stress-induced neural and synaptic atrophy (e.g., of the association cortex and hippocampus) may contribute (McEwen, 1999; Vyas et al., 2002). Excepting (excessive) synaptic pruning (Howes and Onwordi, 2023), it is not typical to think of atrophy as a driver of canalization. Yet, it is likely to play a role in combination with classical mechanisms of acquisition learning, such as LTP (Citri and Malenka, 2007).

Introduced in the late 1940s and inspired by the work of the psychologist Donald Hebb, the “Hebbian postulate” refers to the strengthening (or weakening) of selective neural associations (such as pre- and post-synaptic weights) in response to stimuli. LTP and LTD are two especially well-researched neurobiological mechanisms via which synaptic components (such as post-synaptic density composition and/or neurotransmitter release) are known to change in ways that facilitate the learning of specific associations (Citri and Malenka, 2007). Hebbian mechanisms are core to associative learning, especially acquisition, operant and reinforcement learning, and classical conditioning. In and of themselves, these associative mechanisms are largely agnostic to mental health outcomes, but they *are* fundamental to learning and memory. However, there is evidence that the same basic mechanisms also play a role in the pathogenesis of many mental illnesses, as argued before (Carhart-Harris et al., 2023). In such scenarios, PCP becomes centrally relevant, as does the notion of maladaptive learning, including maladaptive avoidance (Dymond, 2019; Deane et al., 2024; Zeifman et al., 2020), and learned helplessness (Maier and Seligman, 2016).

In what follows, we critique some problematic conventions within neuroscience, namely: (1) using “plasticity” as shorthand for what should properly be called “neuroplasticity”; (2) unconditionally treating the activation of neuroplasticity markers or mechanisms, such as LTP or synaptogenesis, as examples of “plasticity” when the relevant mechanisms tend to favor the opposite outcome, that is, phenotypic canalization (whether pathological or otherwise); and (3) unconditionally inferring mental-health benefits from the activation of any marker of neuroplasticity. Regarding this third point, the construct of “maladaptive plasticity” is relevant (Nava and Röder, 2011). Previous studies have established links between maladaptive plasticity and a wide range of conditions, such as tinnitus (Shore et al., 2016), pain (Flor, 2008), chronic stress (Radley et al., 2015), and substance use disorders (Nava and Röder, 2011; Kalivas and O'Brien, 2007).

More constructively, we identify a small number of candidate neural biomarkers for the induction of PP, where phenotypic plasticity is maintained. In this regard, phenotypes of core interest include psychological states and traits directly relevant to mental health. We refer to candidate biomarkers of PP processes as “mediational and recalibrative plasticity” (MR-P) markers. Unlike PCP, MR-P markers index processes that support phenotypic plasticity during and after a plasticity window. Somewhat related constructs include “plastic compensation” (Morris and Rogers, 2013)—that is, adaptive plasticity overcoming maladaptive plasticity, “allostatic recalibration” (Lawrence et al., 2026), early-life plasticity or critical periods (Carhart-Harris and Nutt, 2017), “restorative plasticity” (Stahnisch and Nitsch, 2002), and (broadly speaking) “homeostatic plasticity” (Kavalali and Monteggia, 2022). However, none of these fully capture the phenomenon we wish to describe, hence our introduction of MR-P. Table 1 highlights definitions of key terms, and a more comprehensive working glossary and taxonomy of key terms is provided as Supplementary Table S1.

**Table 1:** Working definitions of key plasticity-related terms.

Term	Working definition
Plasticity proper (PP)	Plasticity in its basic dictionary sense as changeability, malleability, or the capacity of a phenotype (including a property of a state or system) to be shaped or molded.
Neuroplasticity	An induced change in brain structure, function, activity, connectivity, synaptic organization, or neural dynamics.
Phenotypic plasticity	The capacity of an organismal phenotype to vary in response to environmental or contextual conditions.
M-plasticity	Mediational plasticity: an acute state and its measurable markers associated with increased phenotypic plasticity.
R-plasticity	Recalibrative plasticity: downstream movement away from phenotypic canalization and toward restored flexibility, balance, integration, or adaptive responsiveness.
MR-P	Mediational and recalibrative plasticity: the compound process by which the upstream occurrence of M-plasticity favors a downstream occurrence of R-plasticity.
PCP	Paradoxically canalizing plasticity: neuroplastic, learning, or adaptation processes that culminate in reduced phenotypic changeability, that is, phenotypic canalization.
Canalization	Reduction of phenotypic variability into a more constrained and unchanging configuration.
Allostatic recalibration	Allostasis is “stability through change”; allostatic recalibration is a shift in homeostatic/regulatory set points back to a position of relative flexibility.

## 2 Plasticity as changeability

Adopting the definition of phenotypic plasticity as the *changeability* of a phenotype, then, in the context of neuroscience, it follows that a neural phenomenon (e.g., a state with a particular neurophysiology and neurochemistry) could be considered a marker within a process of increased phenotypic plasticity. Further, we can differentiate between a mediational type of neural phenomenon (M-plasticity) and a subsequent recalibrative one (R-plasticity)—with phenotypic plasticity being elevated in association with both.

Regarding mediation, we can consider parallel psychological and neurobiological phenomena, such as increases in the entropy of spontaneous brain activity (ESBA) and an expansive quality of phenomenal consciousness (Carhart-Harris, 2026), characterized by heightened emotional sensitivity and release (Roseman et al., 2019) and psychological insight (Peill et al., 2022). We can also consider sub- or post-acute improvements in outlook, meaning-in-life (Roseby et al., 2025), and well-being (Lyons et al., 2026) with parallel changes in the flexibility (if not plasticity) of brain network dynamics (Lyons et al., 2026; Daws et al., 2022).

We can consider interventions or experimentally manipulated conditions as “predictor variables,” an acute neurophysiological marker as a “mediator,” contextual conditions as “moderators” (Hayes, 2022), and the subsequent sub- or post-acute phenotypic change, for example, a mental-health change, as the main dependent variable. In some cases, the relevant process would qualify as “moderated mediation,” that is, the intervention (e.g., *Drug* × *Supportive context combination*) influencing downstream (e.g., mental health related) outcomes through acute M-plasticity and later recalibration, while context guides the direction of those mediated effects. From the perspective of mental-health research, the dependent variable of most interest will be a psychological or clinically relevant functional outcome, such as a generic measure of mental health (e.g., wellbeing), or an assessment of symptom severity. Other relevant health outcomes include psychological flexibility, quality of life, social or occupational functioning, service use, and the frequency of risky alcohol or other substance use.

Thought of in this way, mediation and moderation explain an interaction between a catalyst (e.g., a pro-plasticity drug action) combined with contextual conditions guiding relevant phenotypic changes, such as sub- or post-acute changes in mental health outcomes. The changeability of psychological phenotypes, including states or traits, is upregulated by the activation of M-plasticity (e.g., via a pro-plastic drug effect). Still, the eventual direction of change, that is, improved or worsened mental health, depends on the interaction between the induced M-plasticity and surrounding conditions. In the sections that follow, we elaborate on this point by discussing ESBA as a specific candidate example of a brain-based causal mediator of phenotypic plasticity.

We also introduce a second, complementary component of the M-plasticity construct, which we call “recalibrative plasticity” or “R-plasticity.” We refer to the two complementary types of plasticity in combination as “mediational and recalibrative plasticity” or “MR-P,” and we refer to markers of this type of plasticity as “MR-P” markers. The R component of MR-P sets a condition on M-plasticity: it says that for M-plasticity to be aligned with PP, the sub- or post-acute phenotypic change must maintain or promote phenotypic plasticity. The prefix “re,” which is Latin for “again,” “back,” or “anew” is commonplace in terms pertaining to a consistent and relevant theme, for example, restore, repair, reset, recalibrate, remediate, remedy, regenerate, renew, recover, revert.

While we single out “recalibrative” as the “R” in “R-plasticity,” alternatives could have been picked from the list above. However, by choosing “recalibrative” we wish to convey the property of moving away from canalization and *back* toward a state of relative plasticity, where the relevant phenotype is more environmentally sensitive and able to change. It does not become rigid, narrow, or unchanging.

Activation of M-plasticity alone is insufficient to favor downstream improvements in mental health unconditionally. Still, we maintain that activation of MR-P tends to favor plasticity over canalization (Peill et al., 2024). While M-plasticity entails an upstream brain state that (tends to) predict a downstream increase in phenotypic plasticity, this causal relationship is not unconditional. Rather, directionally positive mental health changes depend on the M-plasticity interacting with environmental conditions that are suitably conducive to the restoration or maintenance of PP. More explicitly, those conditions should not drive or impose specific associations nor (necessarily) interrupt ones that arise spontaneously (Timmermann et al., 2022). Rather, they should be supportive but non-directive, conducive to natural healing mechanisms (Timmermann et al., 2022), analogous to how intrinsic physical healing can be encouraged (Peill et al., 2024). Here, “non-directive” does not mean passive or unstructured; rather, it refers to an approach that avoids imposing specific meanings or associations (Johnson et al., 2008).

If this endogenous, self-organizing process is permitted to occur, the more complete, compound phenomenon, *MR-P*, entails a neuro-psychological reset or recalibration that reflects a more enduring quality of phenotypic plasticity, both *now* and going forwards (Lawrence et al., 2026). Indeed, this is what is implied by “recalibrative.” The notion of allostasis, that is, stability through change, is relevant here, particularly where recalibration restores flexible regulation (Lawrence et al., 2026; Brouwer and Carhart-Harris, 2020). Readers should recognize that R-plasticity is directionally specific; it implies a movement in a restorative direction, that is, away from induced canalization and back toward an earlier, more flexible biological and psychological presentation, consistent with maintained or recovered phenotypic plasticity.

In what follows, we focus on one specific neural example of M-plasticity (ESBA), as well as some other candidate MR-P markers. Our neural account of MR-P describes—not only a brain-state mediator of a downstream increase in phenotypic or psychological plasticity—but also a cascade of structural and functional brain changes that align with downstream increases in psychological plasticity. Concrete examples of psychological plasticity include increased openness (Erritzoe et al., 2019), psychological flexibility (Close et al., 2020), acceptance (Close et al., 2020; Watts et al., 2017), absorption (Ott et al., 2005), susceptibility (Belsky and Pluess, 2009), sensory sensitivity (Greven et al., 2019), emotional responsivity (Kaelen et al., 2015), suggestibility (Carhart-Harris et al., 2014a), connectedness (Watts et al., 2022), and equanimity (Rogers et al., 2020). Furthermore, increases in the context sensitivity and general *changeability* of a generic index of mental health, such as wellbeing, are also consistent.

According to our framework, the neural component of MR-P signals a *potential for change*, consistent with PP. One could conceive of a scenario, however, in which a brain change is activated in the absence of subsequent enduring psychological or behavioral change—akin to insight or contemplation that has not yet translated into action. In such a scenario, one could say that only one component of MR-P has occurred, that is, the upstream component—M-plasticity. Yet, the downstream change—R-plasticity—has failed to manifest, whether due to habituation or a ceiling effect (e.g., because a relevant change has already occurred) or the combination of M-plasticity with unfavorable conditions. In the worst of scenarios, M-plasticity combined with psychological vulnerability and adverse conditions could drive PCP and associated psychopathology (Brouwer and Carhart-Harris, 2020; Bremler et al., 2023), as we discuss in Section 4.

To aid readers’ comprehension of the mechanistic framework being expounded here, consider the metaphor of heat being applied to a metal to enhance its plasticity, that is, its *changeability* (or *malleability*). In this metaphorical context, the metal can be regarded as the phenotype, such as a mental-health outcome, and heat can be regarded as the mediating brain state (M-plasticity). A closely related model has been proposed before (Carhart-Harris et al., 2023), with some empirical validation (Ruffini et al., 2023). We can also extend this metaphor to include the recalibrative (R-plasticity) component of MR-P, where post-heating cooling permits recalibration of the crystalline structure—or atomic grain—of the metal alloy, away from compositional inhomogeneity and toward greater atomic homogeneity, uniformity, or balance.

In metallurgy (the science and technology of metals), the process of heat-driven recalibration is known as “annealing.” The cycle of heating and slow cooling promotes a more ductile and durable metal, less prone to brittleness and breakage. Indeed, this metallurgical phenomenon inspired an analogous phenomenon in computing, known as “simulated annealing,” where the introduction of “noise” or a temperature function within an in silico (dynamic) computational system can help it avoid getting stuck in repetitive cycles (attractors) while also helping it to escape any such canalized states—should they occur (Carhart-Harris et al., 2023).

Psychologically, the recalibrative, or annealing-like, process would be comparable to realizing a more integrated psyche; in this respect, the notions of “individuation” (Carl Jung) (Jung, 1967) and “holotropism” (Stanislav Grof) are relevant (Grof and Bennett, 1993). Thus, the heat-mediated movement toward compositional uniformity or balance offers a useful analogy for MR-P: a mediating brain state (M-plasticity) and recalibrative brain change (R-plasticity) that favors the emergence of a more flexible psychological state and set of traits (PP), conducive to mental health.

## 3 Measuring and testing MR-plasticity (MR-P)

In the context of neuroscience, psychology, and psychiatry, we could test for, and potentially validate, the occurrence of MR-P by measuring the rate, size, variability, and/or context-dependency of a subsequent phenotypic change. For example, we might hypothesize a hypersensitive psychological state (acute) and a change in a generic aspect of mental health, such as wellbeing (a sub- or post-acute change). Furthermore, we might aim to test this under two conditions: one in which a specific brain change is hypothesized to occur, and one in which no such brain change is expected to occur, with the latter serving as a control or placebo condition. M and R plasticity are complementary and interrelated, that is, the occurrence of M-plasticity tends to favor R plasticity. However, to address accusations of circularity, markers of M and R plasticity should be stipulated *a priori*. In sections 6–8, we describe specific MR-P markers. Table 2 lists how markers can be tested and potentially validated. We also address how evidence could weaken or falsify the MR-P model.

**Table 2:** Operational criteria for candidate MR-P processes.

Construct	Required evidence	Timeframe	Interpretation if absent or mismatched
M-plasticity	Pre-specified acute marker of increased phenotypic plasticity, including enhanced context-sensitivity. Prime candidate = ESBA (Carhart-Harris, 2026).	Acute/session level	If absent, the specific candidate M-plasticity marker is not supported for that intervention or context.
R-plasticity	Pre-specified downstream movement away from canalization toward maintained or increased phenotypic plasticity, for example, synaptic rescue (Moda-Sava et al., 2019) and/or network desegregation or global integration (Daws et al., 2022), correlating with psychological plasticity.	Sub-acute to long term	If absent after M-plasticity, classify as M-plasticity without R-plasticity, and therefore not full MR-P.
MR-P	Evidence that M-plasticity precedes and predicts R-plasticity, for example, ESBA predicting synaptic rescue or increased global integration.	Acute-to-longitudinal	If M-plasticity does not predict downstream R-plasticity and/or an increase in phenotypic plasticity, despite the presence of a supportive context for this, then the MR-P model is weakened or falsified.
PCP/negative canalization	Neuroplastic change associated with behavioral narrowing or rigidity.	Acute-to-longitudinal	If present, classify the process as PCP rather than MR-P.
Null/mixed response	No marker change, no enduring increase in phenotypic plasticity, or inconsistent outcome pattern.	Any relevant timeframe	Classify as null, mixed, or indeterminate evidence that weakens the MR-P model.

Demonstrating that the specific direction of a phenotypic change is context-dependent, that is, it is dependent on the environmental conditions in which M-plasticity takes place (i.e., a *moderated mediation*) (Hayes, 2022), would meet a defining criterion for PP. However, as explained earlier, the mere activation of M-plasticity/changeability does not pre-determine what *actual* change will occur. This is analogous to heating a metal but not shaping it, or shaping it haphazardly, once it becomes malleable. The activation of changeability merely *implies* a *potential for change*.

However, if we manipulate the conditions or *context* in which M-plasticity arises, we could encourage a particular type of change. For example, an M-plasticity-promoting drug, such as a psychedelic (Carhart-Harris, 2026), could be thoughtfully paired with supportive environmental conditions, such as non-directive compassionate care (Timmermann et al., 2022) and music-listening (Carhart-Harris et al., 2018), to bias a positive mental-health change.

**Figure 1:**
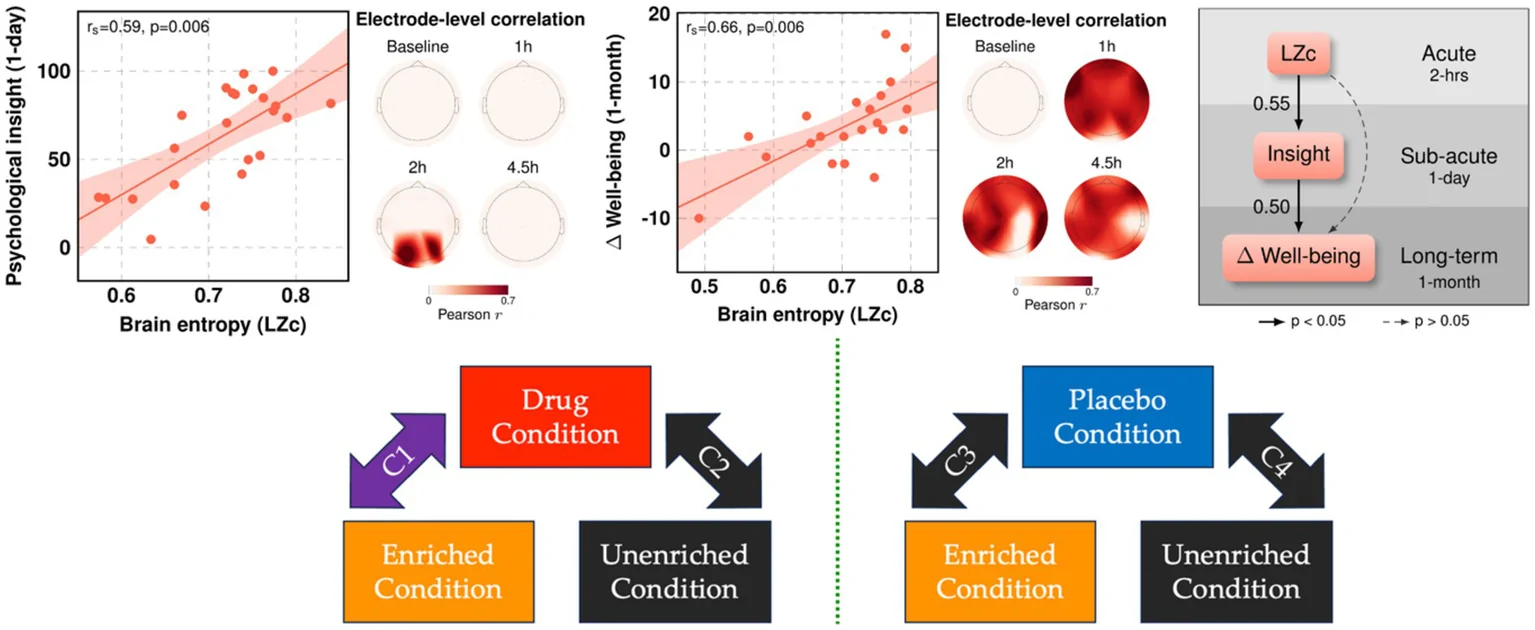
Increased ESBA predicts sub-acute psychological insight (left) and 1-month improvements in wellbeing (top, center). Top row adapted from Lyons et al. (2026), licensed under CC BY 4.0 (https://creativecommons.org/licenses/by/4.0/); bottom-row 2 × 2 design revised from Carhart-Harris et al. (2018). A mediation model (top, right) shows psychological insight as a mediator of the association between ESBA, indexed by Lempel-Ziv complexity (LZc), and 1-month improvements in wellbeing. The bottom row illustrates a 2 × 2 factorial design for testing a putative interaction between a *pro-plastic drug* (e.g., a psychedelic) and *context*. C1 (purple), combining drug administration with enriched context, is hypothesized to be the condition most conducive to mental-health improvement.

A rigorous two-by-two factorial design that experimentally manipulates *context* could be employed. Theoretically, a design like this could provide a clear demonstration of the hypothesized context-dependency (*moderator*) of phenotypic change (*dependent* var*iable*) facilitated by the activation of M-plasticity (*mediator*). See Figure 1, bottom, for an illustration of this design.

In such an experiment, we would hypothesize that M-plasticity induction would produce a more volatile, labile, or sensitive mental state (Brouwer and Carhart-Harris, 2020; Wang et al., 2024), conducive to change. Demonstrating heightened environmental/context sensitivity during heightened M-plasticity would offer a compelling test—and potential validation—of the relevant assumptions regarding M-plasticity (Greven et al., 2019).

For example, psychedelic therapy might be hypothesized to activate both M- and R-plasticity (MR-P) (Carhart-Harris et al., 2023). In a between-subjects design, half of a sample could receive a psychedelic, whereas half could be given an inert placebo. We can call this two-level factor the “drug factor.” The two levels are *drug,* for example, 25 mg of psilocybin, and *placebo,* for example, an inert capsule or tablet. We can also experimentally manipulate a second factor, which we will call the “context factor.” For simplicity, we will also give this (moderating) factor two levels, namely: (1) a positive or *enriched* context (mimicking the “therapy” component of psychedelic therapy) and (2) an *unenriched* context, devoid of any overtly supportive elements, such as compassionate care, access to music-listening, relaxation-promoting stimuli, and comfortable furnishings for the dosing session.

A brief note on safety and ethics: an unenriched condition implies no active enrichment, for example, participants are dosed in a standard-of-care medical room. Careful screening, informed consent, an appropriate briefing, trained session monitoring, and basic comfort would be retained to ensure procedures are sufficiently safe and ethical. Moreover, staff would be trained in standard operating procedures for responding to acute or protracted distress, that is, with various de-escalation techniques, including the use of rescue medication in extreme scenarios (Johnson et al., 2008).

In such a design, the primary aim would be to test for a hypothesized *Drug* × *Context interaction* (Poggini et al., 2021). To help explain the main hypothesis, we will give an example of a hypothesized result. Imagine substantial variation in psychological responses in the two psychedelic drug conditions (*enriched* and *unenriched*). More specifically, imagine that, on average, very positive psychological responses are seen in the *Psychedelic* × *Enriched* condition, whereas less positive (or more negative) psychological responses are seen in the *Psychedelic* × *Unenriched* condition, with high response variance across the two psychedelic conditions combined. In contrast, consider the two placebo conditions (*enriched* and *unenriched*). Because placebo should not promote phenotypic plasticity, we would predict a tighter spread of responses (less statistical variance) across the two placebo conditions (*enriched* and *unenriched*), reflecting weaker context-dependency.

Continuing the theme of experimentally testing MR-P, another potentially important variable could be referred to as individual differences in “inherent plasticity” or “trait plasticity.” For example, relevant genetic differences (Pluess et al., 2010), varying life experiences during sensitive or critical periods marked by heightened plasticity (including neuroplasticity) (Dehorter and Del Pino, 2020), and previous experience with pro-plastic compounds, such as psychedelics (Nardou et al., 2023), might contribute to differences in trait-level inherent plasticity at baseline, prior to any experimental intervention, analogous to a metal’s inherent ductility or brittleness prior to annealing. The notion of differential susceptibility is relevant here (Belsky and Pluess, 2009; Pluess et al., 2010). Individual differences in inherent plasticity (e.g., trait suggestibility or absorption) could be included as a model variable in a predictive model. This variable may explain group-level differences downstream (Ott et al., 2005), such as differential sensitivity to a pro-plastic intervention, such as a psychedelic drug (Ott et al., 2005), and its interaction with a specific contextual ecology (Lawrence et al., 2026; Timmermann et al., 2022).

Continuing with the metallurgical analogy, we can consider two salient factors for predicting M-plasticity induction: (1) heat (e.g., a drug-induced state of M-plasticity) and (2) the intrinsic ductility or brittleness of the metal (e.g., the individual’s inherent plasticity). For example, within certain bounds, we might hypothesize that higher temperatures and more ductile, less brittle materials confer greater malleability. As just addressed, a key critical factor is the *context* in which the heightened evoked plasticity occurs. Within the metallurgical analogy, *context* can be thought of as the application of work or force to the heated material. To help illustrate this, imagine heating an already ductile metal so that residual stress is relieved and the material becomes more workable. If the material is then shaped under intentional and controlled conditions, one could steward a desired outcome. Still, if the same (heated) substance was subjected to haphazard force, one could not reliably expect this.

In many cases, popular neuroplasticity markers measure mere *change,* analogous to an already shaped metal. They do not always measure *changeability,* analogous to a heated, more workable metal. Worse still, many neuroplasticity markers favor *phenotypic narrowing*, the process of canalization, in which phenotypic and psychological changeability become *less likely* rather than more likely. This is what we refer to as *paradoxically canalizing plasticity* (PCP), which means phenotypic plasticity declines. One could argue that many putative examples of neuroplasticity are *incomplete*, or at worst, *misleading* examples of PP.

In summary, the MR-P framework yields a clear experimental prediction. If enhanced M-plasticity is directionally agnostic, then we might expect greater variation in responses in the *Psychedelic × Unenriched* condition (see C2 in Figure 1) vs. the *Placebo × Unenriched* condition (see C4 in Figure 1), but no consistent directionality to the change. In such a condition (see C2 in Figure 1), we would assume that the drug promoted *changeability*/*plasticity*, but highly varied and unpredictable responses followed from the absence of contextual structure or guidance. Such an outcome would lend further support to the principle (Carhart-Harris et al., 2018) and evidence (Syed et al., 2026; Florineth et al., 2026; Rich et al., 2025; Roseman et al., 2024; Farinha-Ferreira et al., 2025) that responses to psychedelic medicines are essentially *context dependent*. Relatedly, atypical null (efficacy) findings in psychedelic clinical trials could partly be explained by a deviation from standards of best practice for psychedelic therapy—and more specifically, the requirement for supportive care (Johnson et al., 2008).

## 4 Matters of safety and risk

It seems likely that some individuals are at elevated risk of adverse responses to psychedelics, analogous to the notion of “brittleness” in metallurgy. For example, individuals with a history of personality disorder or psychotic disorder appear to have more volatile (i.e., less reliably favorable) responses to psychedelics (Marrocu et al., 2024). There may be an important distinction to make here between inherent plasticity conducive to drug-induced M-plasticity and characterological brittleness signaling an elevated risk of psychological decompensation through M-plasticity. Related study on attachment insecurity and responses to psychedelic therapy is relevant here (Stauffer et al., 2020). If pro-plastic agents, such as psychedelics, are to be developed clinically, it will be critically important to identify how to screen for traits associated with elevated risk effectively.

Theoretically, M-plasticity-promoting interventions may require special caution in individuals with the following vulnerabilities: psychosis-spectrum, bipolar-spectrum, severe dissociation, active suicidality, severe substance-use disorder, or high social instability. In such cases, increased phenotypic plasticity may not translate into adaptive recalibration and may instead risk destabilization, crisis, and maladaptive meaning-making. These considerations underscore the importance of careful patient selection, preparation, acute session monitoring, integration, follow-up, and relapse-prevention planning when developing M-plasticity-promoting interventions for clinical populations (Johnson et al., 2008).

These concerns are consistent with studies on self-illness ambiguity, where patients may struggle to distinguish their own intentions, feelings, identity, or agency from the effects of illness or diagnosis (Dings and Golova, 2026; Crippen, 2025). In cases where reality testing is impaired, lower-demand forms of support may be required before insight-oriented or emotionally intensive work can safely occur. For contact-impaired clients, including those with psychosis-spectrum vulnerabilities, “pre-therapy” may be beneficial (Prouty et al., 2002). Psychological openness is not inherently therapeutic. Sufficient ego-integrity, relational support, and integration are required for an opening process to be sufficiently well-tolerated and ultimately beneficial.

## 5 Cases of paradoxically canalizing plasticity (PCP)

What are some concrete examples of markers of PCP? Focusing on anatomical neuroplasticity, anatomical changes—of almost any kind—lend themselves to concrete measurement and illustrative display; for example, they can serve to communicate an appealing narrative. For example, images of increased dendritic branching after drug exposure, such as psychedelic exposure, appeal to our intuitions regarding an intervention’s functional implications, and medicinal potential. However, if we are faithful to the meaning of PP as *changeability*, then increases in markers of anatomical neuroplasticity can only be regarded as properly aligned with PP if we can confirm that these candidate markers imply greater *adaptive* changeability going forward.

For example, one could infer that psychostimulant-induced increases in excitatory synaptic input onto ventral tegmental area (VTA) dopaminergic neurons (Ungless et al., 2001), or rapid synaptogenesis/spinogenesis within the nucleus accumbens after cocaine exposure (Dos Santos et al., 2017), reflect structural and functional changes catalyzed by exposure to the relevant drug (Dos Santos et al., 2017; Ungless et al., 2001). Certain psychostimulants, such as cocaine and methamphetamine, have high abuse and addiction potential (Dos Santos et al., 2017; Ungless et al., 2001). Psychostimulant-induced spinogenesis within the nucleus accumbens reflects an anatomical brain *change* related to (maladaptive) learning (Kalivas and O'Brien, 2007; Hyman et al., 2006), and not enhanced *changeability* as presented by our MR-P framework. More concretely, an individual who repeatedly uses cocaine and develops cocaine dependence will have undergone a process of multi-domain canalization. In such a scenario, we can say that cocaine exposure caused a neuroplastic effect aligned with PCP but opposed to PP.

Long-term potentiation is a classic synaptic marker of Hebbian plasticity; indeed, LTP may be the most researched subtype of neuroplasticity within neuroscience. LTP indexes the strengthening, for example, potentiation, of synaptic transmission between neurons via repeated activity (Dolphin et al., 1982). Hebbian neuroplasticity is typically homosynaptic or input-specific, as opposed to heterosynaptic neuroplasticity. Homosynaptic neuroplasticity refers to specific synaptic components becoming potentiated, as opposed to a more generalized change in synaptic weights (Chistiakova et al., 2014). Homosynaptic neuroplasticity consolidating maladaptive associations is aligned with PCP, whereas more distributed forms of neuroplasticity may be more compatible with adaptive phenotypic recalibration (Moda-Sava et al., 2019).

The popularity of Hebbian neuroplasticity can be partly explained by its amenability to testing, which has traditionally occurred in preclinical models, although functional proxies have also been proposed in humans (Sumner et al., 2020). Hebbian neuroplasticity is relevant to experience- and context-dependent learning; it offers an intuitively appealing heuristic for understanding important aspects of brain, mind, and behavior, especially as these relate to learning.

However, examples of Hebbian neuroplasticity have often been leveraged to make generalized claims regarding the induction of “plasticity,” thereby risking an overgeneralization fallacy. In such scenarios, a specific measure, for example, an assay of LTP in a mouse brain or slice, may be treated as evidence of something more general, such as enhanced psychological plasticity (and therefore therapeutic potential) in humans. Compounding the problematic nature of this custom, Hebbian neuroplasticity does not just misalign with plasticity proper; it may even counteract it. In the worst cases, such as the development of drug addiction, canalization—underpinned by Hebbian mechanisms—may be critical to the instantiation of the pathology, as laid out in the “canalization model of psychopathology” (Carhart-Harris et al., 2023). Briefly, this model proposes a key role for adversity and the canalization of phenotypes deemed maladaptive by social consensus in the pathogenesis of mental illness, and it forms part of the foundational logic of the MR-P framework laid out here.

## 6 Psychedelics as promoters of mediational and recalibrative plasticity (MR-P)

Psychedelics are promising experimental medicines within mental health research (Nutt and Carhart-Harris, 2021). It has become customary to imply, whether implicitly or explicitly (Vargas et al., 2021), that mental-health improvement can be inferred from psychedelic-induced increases in markers of neuroplasticity. Critically, this inference has often rested on evidence of cortical spinogenesis (Olson, 2018). Some have categorized compounds based on their ability to increase cortical spinogenesis, calling them “psychoplastogens” (Olson, 2018), despite the neuroanatomical nature of the phenomenon. Etymologically, “psyche” carries meanings of soul, mind, spirit, and life, with later psychological usage referring to the totality of the conscious and unconscious mind (Ostrand et al., 2025). Thus, for compounds defined only by neuroanatomical effects, such as cortical spinogenesis, it would be more accurate to use the term “neuroplastogen,” unless, of course, there is evidence that these drugs can truly promote plasticity proper (PP), which they may well do, especially when (properly) delivered as context-dependent medicines.

## 7 Candidate markers of R-plasticity

Examples of anatomical neuroplasticity, such as cortical spinogenesis or synaptogenesis, are typically shown in preclinical preparations, including *in vitro* (Ly et al., 2018), *ex vivo* (Nardou et al., 2023), and *in vivo* (Shao et al., 2021) models. Translating from something as specific as preclinical cortical spinogenesis to a meaningful human homolog relevant to mental health is not a trivial step (Carhart-Harris, 2023a). Even if an effect like spinogenesis in rodent brain tissue does reliably translate to something homologous in a living human brain and genuinely reflects enhanced psychological *changeability*, can we feel confident that mental-health improvement should naturally and unconditionally follow? Interestingly, ketamine has been shown to rescue stress-related prefrontal spine and circuit dysfunction (Moda-Sava et al., 2019), while psilocybin has been shown to promote hippocampal neuroplasticity relevant to fear extinction in mice (Du et al., 2023). However, translating these apparent examples of restorative plasticity to human brains, behavior, and experience remains an underdeveloped (if not entirely missing) link. That said, unlike LTP, increased (heterosynaptic) spinogenesis in the cortex does more naturally imply increased PP; for example, it may suggest a potential substrate of restorative plasticity and allostatic recalibration (Lawrence et al., 2026).

Some potentially relevant human homologs of functional recalibration (and restorative plasticity) have been proposed via neuroimaging (Lyons et al., 2026; Daws et al., 2022; Deco et al., 2024). Briefly, candidate biomarkers consistent with functional recalibration after psilocybin therapy for depression (compared with the SSRI escitalopram) include decreased brain network modularity or increased global integration (Daws et al., 2022), reconfigured hierarchical organization (Deco et al., 2024), and relatively preserved or enhanced neural sensitivity to social or emotional stimuli (Wall et al., 2025). One way to qualify whether a change in brain function is consistent with R-plasticity is to ask whether the change reflects a return to an earlier configuration, prior to canalization. This is the case with global integration (Fair et al., 2007) and synaptic rescue (Moda-Sava et al., 2019), for example.

Procedures for delivering psychedelic medicines within clinical trials and beyond currently follow a psychology-centric paradigm broadly consistent with the methods and reported outcomes of psychotherapy. The occurrence of “emotional release” (Roseman et al., 2019) and “psychological insight” (Peill et al., 2022), as well as “mystical type” (Ross et al., 2016) or “peak experiences” (Barsuglia et al., 2018), has been found to predict variance in therapeutic outcomes across trials, including in a relatively large phase 2 trial of psilocybin therapy (Goodwin et al., 2025). Identifying relevant biomarkers of psychedelic-induced brain changes and changeability will contribute valuable data of relevance to the validity of the MR-P framework, as outlined here. Such biomarkers may also elucidate how relevant biological and psychological theories converge or diverge, potentially helping to reconcile across these domains (biology and psychology) in a meaningful and productive way (Lyons et al., 2026)—moving us toward a more integrative, evidence-based consilience of paradigms.

For example, the reopening of a critical period for social reward learning has been proposed as a model of the action of psychedelic medicine (Nardou et al., 2023). This process-based model is broadly consistent with and complementary to the MR-P framework outlined here, whereby an upstream brain effect leads to downstream neurobiological and psychological changes consistent with PP—albeit when paired with appropriate contextual support. This critical-period model is based on preclinical research (Nardou et al., 2023; Edsinger and Dölen, 2018). Still, it raises the possibility of homologous mechanisms in humans receiving psychedelics [or psychedelic-like substances, like 3,4-methylenedioxymethamphetamine (MDMA) (Sottile and Vida, 2022)] as part of psychedelic therapy for the treatment of mental illness. Indeed, the parallel with developmental plasticity windows, and more broadly with post-stroke plasticity windows (Dromerick et al., 2021), is instructive. In each case, a bounded temporal period of heightened plasticity has relatively clear endpoints and depends strongly on contextual input in shaping outcomes that may be considered adaptive or maladaptive.

Early life is characterized by (critical) periods of heightened plasticity (including neuroplasticity), during which experience can exert especially strong effects on learning, development, and social adaptation. The transient recovery or reopening of such critical-period-like qualities in adulthood may be relevant to the promotion of PP via psychedelics. Relevant examples include increasing psychological flexibility (Close et al., 2020) and social functioning (Watts et al., 2017). The link between the rescue or replenishment of atrophied cortical neuronal spines (Moda-Sava et al., 2019) following adversity-related stress (Moda-Sava et al., 2019) may well be relevant to the therapeutic action of psychedelics in humans (Carhart-Harris et al., 2023; Siegel et al., 2026). Further, brain-slice electrophysiological signatures of neuroplasticity observed in preclinical tissue (Nardou et al., 2023; Kramer et al., 2025) could relate to homologous electrophysiological signatures in humans, as per the ESBA model. These would be candidate markers of M-plasticity. However, we should caveat this by acknowledging the challenge of extrapolating from animal behavior or physiology to human psychology (Carhart-Harris, 2023a).

Having considered psychedelics as candidate promoters of MR-P, we now turn to ESBA as a candidate marker of M-plasticity and a possible bridge between psychedelic neurophysiology, subjective experience, and downstream psychological change.

## 8 ESBA as a candidate marker of M-plasticity

**Figure 2:**
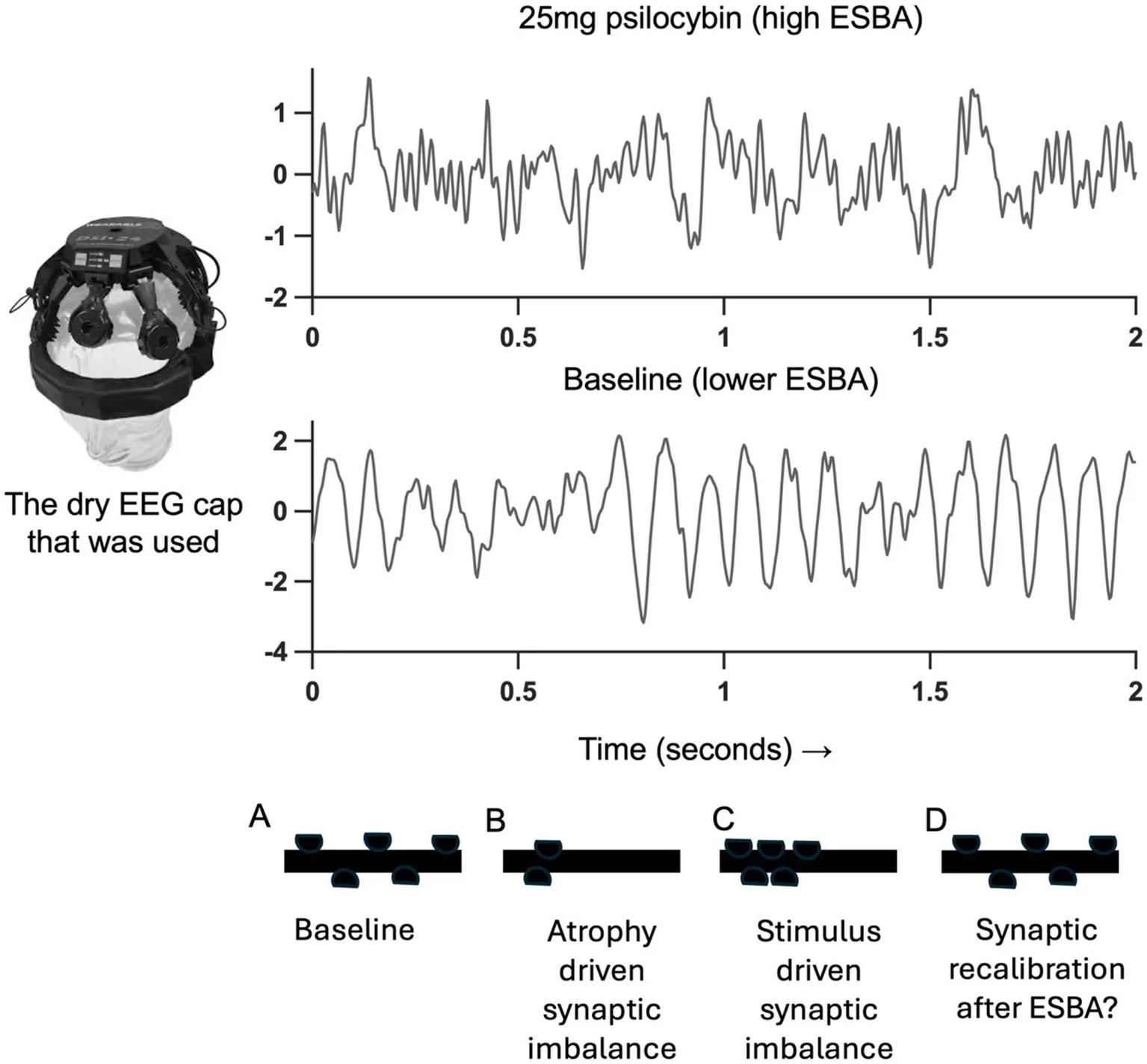
An example of an entropic time series recorded via EEG under 25 mg psilocybin (upper trace) vs. a drug-free baseline condition (lower trace) in the same healthy individual. These data are from a recently completed but unpublished study (Carhart-Harris, 2023b) and are included here as a single-subject illustrative example. The example is consistent with the well-established phenomenon of increased signal complexity or entropy under psychedelic drugs, calculated from EEG- or MEG-recorded potentials during resting-state conditions, such as quietly relaxing with eyes closed (Schartner et al., 2017). The *y*-axes for the potentials shown in the upper two traces are scaled by 10^−5^ V. There is tentative evidence that the psychedelic-like dissociative ketamine (Moda-Sava et al., 2019), and the canonical psychedelic, psilocybin (Du et al., 2023), may exert recalibrative actions at the synaptic level. It is currently unknown whether ESBA plays a role in driving this effect. The lower schematic illustrates our hypothesis (D) that psychedelic administration, paired with a psychologically supportive and enriched context, could rescue and recalibrate synaptic inhomogeneities, potentially through neural mechanisms indexed by increased entropy of spontaneous brain activity (ESBA). Such inhomogeneities or imbalances could have been driven by stress-induced atrophy or pruning (B) as has been shown across species (Carhart-Harris et al., 2023; Dias-Ferreira et al., 2009), and/or Hebbian-related synaptic restructuring (C) akin to what can occur with chronic psychostimulant exposure (Dos Santos et al., 2017; Ungless et al., 2001). Images (A–D) are intended to represent synaptic spines protruding from a dendrite.

Over the last decade, the entropy of spontaneous fluctuations in population-level scalp or cortical potentials, recorded via electroencephalography (EEG) or magnetoencephalography (MEG) during eyes-closed, task-free resting conditions, has emerged as a promising biomarker of psychedelic action—as well as a possible biomarker of what we refer to here as M-plasticity (Carhart-Harris, 2026). We refer to this neurophysiological quality as “entropy of spontaneous brain activity” or “ESBA” (see Figures 1, 2). To measure and index this quality, we draw from communication science, in which statistical entropy was developed as an index of (potential) information (Shannon and Weaver, 1949).

The current most popular algorithm for calculating entropy in EEG/MEG data, Lempel-Ziv complexity (LZc), treats it as the *compressibility* of a time series. The logic with this marker is that, by increasing ESBA, a psychedelic increases the amount of potentially available cognitive, affective, and sensory information, which may translate into beneficial and actionable *psychological insight*. If the availability of potential information is increased during a psychedelic state, as indexed by increased ESBA/LZc, and this information is properly processed or integrated, evidence suggests that the resulting psychological insight may foster improvements in mental health (Carhart-Harris et al., 2023; Zeifman et al., 2025; Carhart-Harris and Friston, 2019). Put more plainly, the claim is not that increased ESBA is unconditionally beneficial. For example, see studies of elevated ESBA during organic psychosis induced by tetrahydrocannabinol (THC) (Cortes-Briones et al., 2015) and during functional psychosis (Li et al., 2008). Increased ESBA may index a temporary expansion in available cognitive, affective, and sensory material, which, under supportive conditions, can be harnessed to guide beneficial change.

Prior studies using EEG and MEG have shown reliable increases in ESBA or related neural-complexity measures under psilocybin (Lyons et al., 2026; Schartner et al., 2017), lysergic acid diethylamide (LSD) (Schartner et al., 2017; Murray et al., 2024), N,N-dimethyltryptamine (DMT) (Timmermann et al., 2023; Timmermann et al., 2019), and sub-anesthetic but psychedelic-like doses of ketamine (Schartner et al., 2017; Pascovich et al., 2022). Comparable increases have not been observed with some non-psychedelic psychoactive drugs, such as methamphetamine (Murray et al., 2024), whereas sedative compounds (Schartner et al., 2015) and anesthetics (Zhang et al., 2001) reliably decrease ESBA, often in proportion to reductions in wakefulness or “conscious level.” Evidence that THC increases ESBA is mixed: one study suggests that THC dose-dependently elevates cortical entropy in association with psychotomimetic effects (Cortes-Briones et al., 2015), whereas another found no increase (Murray et al., 2024). Other previous work has spotlighted meaningful correlations between increased ESBA under psychedelics and the signature subjective effects of these drugs, such as increases in the richness of conscious experience (Timmermann et al., 2023). Taken together, these data suggest that ESBA is a sensitive candidate acute-state marker of psychedelic action and a possible marker of M-plasticity that can predict downstream improvements in mental health (Lyons et al., 2026)—if conditions are sufficiently supportive.

A theoretical model known as “the entropic brain hypothesis” (EBH) proposes that ESBA has a meaningful counterpart in the experiential domain (Carhart-Harris, 2026). More recently, ESBA has been proposed to index the *size* of phenomenal consciousness. Unpacking this, states that feature increased ESBA, such as psychedelic states and certain highly absorptive meditation states, known as “the Jhanas,” are felt as being more *expansive* (Carhart-Harris, 2026; Chowdhury et al., 2025). A range of pharmacological and sleep research findings (Pascovich et al., 2022; Abásolo et al., 2015), along with cross-sectional (Altıntop et al., 2022) and longitudinal clinical work in disorders of consciousness (Lei et al., 2022), align with the entropic brain principle (Carhart-Harris, 2018; Carhart-Harris et al., 2014b), as reviewed in Carhart-Harris (2026). Furthermore, a recent study found that low doses of LSD increased LZc in a dose-dependent manner, with the higher LSD dose also producing detectable subjective drug effects (Murray et al., 2024).

Recently, ESBA recorded via EEG during eyes-closed rest within a psilocybin therapy protocol was found to predict improvements in mental health (psychological wellbeing) one month later (Lyons et al., 2026) (Figure 1). This predictive relationship was robust, in the sense that it was evident across most EEG sensors and at all three post-dosing time points, that is, 60, 120, and 270 min post-administration of psilocybin (25 mg). Further, ESBA was both directly and indirectly predictive of improved wellbeing. Regarding the indirect relationship, improved wellbeing could be predicted via a mediation path linking acute increases in ESBA with next-day psychological insight measured using a validated scale (Peill et al., 2022) (Figure 1, top right). This indirect path is mechanistically consistent with the principle that psychedelic therapy entails a process of psychological growth, which is also broadly consistent with the basic principles of psychotherapy (Weiss et al., 2024). It is relevant that many research participants regard a high-dose psychedelic experience as being among the most profound and personally meaningful experiences of their lives, evoking parallels with religious conversions and other such transformative experiences (Brouwer and Carhart-Harris, 2020).

Conveniently, the eyes-closed resting condition is consistent with the default procedure for psychedelic therapy (Johnson et al., 2008). This detail is relevant to the use of ESBA as a candidate predictive biomarker of therapeutic improvement via psychedelic therapy, as it implies that the EEG data required for computing ESBA can be collected in an ecologically valid way, with minimal disruption to standard procedures. Recently, EEG acquisition and ESBA computation in a clinical population, fibromyalgia syndrome, were accomplished without disrupting conventional procedures for psychedelic therapy (Bornemann et al., 2024). The procedure was well-tolerated, and preliminary evidence suggests, as hypothesized, that psilocybin, but not placebo, robustly increases ESBA and that this effect correlates with psychological insight. Recent analytical developments make it theoretically plausible to compute and display ESBA values in real time (Mediano et al., 2023). This could make intelligently guided, real-time dose titration feasible for optimizing response, potentially even in a closed-loop fashion.

We feel compelled to ask: *is increased ESBA under psychedelics an M-plasticity marker*; does ESBA imply enhanced phenotypic *changeability*, a catalysis of the *ease*, *size*, *rate* and/or *context-dependency* of psychological change? In the study described above (Lyons et al., 2026), variance in ESBA under psilocybin was predictive of changes in psychological wellbeing, a generic marker of mental health. Applying the metallurgical analogy again, one could regard the relationship between increased ESBA and ensuing mental-health change as analogous to the role of heat in increasing the workability or changeability of a metal’s structure (Carhart-Harris et al., 2023). See Carhart-Harris et al. (2023) and Ruffini et al. (2023) for two relevant models.

According to our MR-P model, inducing M-plasticity, indexed here by increased ESBA, should enhance the importance and influence of *context,* for example, psychological support and music-listening in psychedelic therapy, thereby biasing change, for example, toward improved mental health (Carhart-Harris et al., 2018). Responses to psychedelics have long been assumed to be exceptionally context sensitive (Carhart-Harris et al., 2018). If valid, this assumption would support the view that increased ESBA is *not* a direct causal driver of positive mental-health change but rather a marker of *psychological plasticity*, that is, a context-sensitive *opportunity for psychological growth*. However, see Peill et al. (2024) for another possibility, that is, that psychedelics initiate a process that is intrinsically biased toward health.

Returning to our metallurgical analogy, the pro-plastic action of a psychedelic, that is, its induction of M-plasticity, could function like heating a metal, rendering it more workable. An enriched, supportive ecology might then facilitate the recalibrative component of MR-P, whereas an adverse context could instead engage PCP. Perhaps an adverse context is analogous to poorly guided hammering of a heated horseshoe: the heat makes the metal workable, but misdirected hammering can deform it away from a functional fit rather than shape it toward one. A scenario like this is analogous to canalization: the driving of a specific morphological change toward a putatively maladaptive endpoint. Conversely, combining M-plasticity with an enriched, supportive context is analogous to annealing; heat renders the metal workable, while controlled conditions allow internal stresses to relax and the structure to become more homogeneous. Understood this way, psychedelic-induced M-plasticity, indexed here by increased ESBA, may culminate in PCP under adverse conditions, whereas the same M-plasticity under supportive conditions is consistent with MR-P and, therefore, plasticity proper.

## 9 The broader meaning of context

We recognize that “context” applies in a broad sense in mental health research; it does not merely denote the immediate sensory or interpersonal features of a psychedelic dosing session. Consistent with biopsychosocial and function-oriented clinical models (World Health Organization, 2001; Engel, 1980), context can be understood as part of a broader biopsychosocial ecology, encompassing interacting biological, psychological, relational, social, and service-level conditions. These factors may include individual differences in drug response, neurodevelopmental vulnerabilities, prior learning, expectancy, therapeutic alliance and the care effect, priming, patient intentions and agency, family or peer ecology, housing and employment, stigma, financial, legal, and institutional constraints, and continuity of care (Miller and Kaptchuk, 2008; Bernstein and Brown, 2017). Thus, psychedelic-induced M-plasticity should be understood as enhancing context sensitivity—which could interact with a person’s broader ecology, that is, the same pro-plastic state (whether acute or extended) may contribute to healthy restoration (R-plasticity) or canalization depending on the wider conditions in which it is embedded (Carhart-Harris et al., 2018; Hartogsohn, 2016).

In relation to psychedelic therapy, *context* in the broader psychosocial sense has been referred to as the “matrix” (Eisner, 1997).

## 10 Expectancy and suggestibility

In line with convention, most psychedelic therapy trials have adhered to the double-blind design. Thus far, the conspicuous subjective effects of psychedelics typically corrupt the integrity of the blind (Orsini et al., 2026), causing a phenomenon known as “functional unblinding.” Be this as it may, it should not be assumed that functional unblinding unconditionally activates an expectancy bias. Indeed, recent evidence has challenged the assumption that expectancy is a cause of positive psychedelic therapy outcomes (Szigeti et al., 2024; Lewis et al., 2025). The correct way to proceed here is to measure and model the influence of expectancy. Only then can we know if it is an important explanatory variable for moderating responses (Carhart-Harris et al., 2014a; Szigeti et al., 2024; Hartogsohn, 2016). Presently, pre-treatment expectancy has functioned as an unusually poor predictor of response to psychedelic therapy (Szigeti et al., 2024).

Suggestibility, on the other hand, appears to have predictive power (Szigeti et al., 2024). One explanation for this is that aberrant prior assumptions (such as low expectations for recovery in depression) are a ripe target for the core action of psychedelic therapy, as per the RElaxed Beliefs Under pSychedelics (REBUS) model (Carhart-Harris and Friston, 2019). In contrast, suggestibility relates to the trait-level inherent plasticity (discussed in section 3) that renders certain individuals more sensitive to the context-dependent action of psychedelic therapy.

## 11 Psychotherapy

Psychotherapy has long been framed as biologically consequential, insofar as learning, memory, emotion regulation, attachment, and behavior are instantiated in brain and body processes (Kandel, 1998). Consistent with this view, psychotherapy has been associated with measurable changes in brain function (Linden, 2006; Barsaglini et al., 2014). Thus, psychedelic therapy can be understood as a catalyst of plasticity-relevant therapeutic and social processes, in which several moderator variables can shape the direction and durability of mental health changes (Lane et al., 2015). In this sense, psychedelic therapy is best conceptualized as a catalyst of a therapeutic process embedded within a broader context of care, rather than a pure pharmacotherapy model or stand-alone replacement for psychotherapy (Greenway et al., 2020).

State-sensitive psychotherapies such as intensive short-term dynamic psychotherapy (ISTDP) and broader experiential dynamic therapies (EDTs) provide useful clinical analogs for psychedelic therapy. These approaches operationalize moment-to-moment attention to affective arousal, defensive responding, tolerance or overload, and in-session emotional processing. Process models of psychotherapy are relevant in this context (Abbass et al., 2012; Hoviatdoost et al., 2020; Lilliengren et al., 2025). State-sensitive psychotherapies are also highly relevant because they offer a framework for informing when to deepen emotional processing or pause and defer it. See also Zeifman et al. (2023).

## 12 Is the “trip” necessary?

Despite debate (Olson, 2020), the weight of the evidence favors the position that the psychedelic experience itself, that is, the subjective experience during dosing, predicts outcomes from psychedelic therapy (Lyons et al., 2026; Nautiyal and Yaden, 2022). This bears relevance to the so-called “*non-psychedelic psychedelic*” debate (Carhart-Harris, 2023a), that is, the hypothesis that psychedelic subjective effects may be an undesirable and non-essential (e.g., epiphenomenal) component of the therapeutic action of psychedelics (Olson, 2020). Due, in part, to problematic translational assumptions, we remain skeptical that any preclinical (Olson, 2020) or case evidence (Rosenblat et al., 2023) suggestive of this hypothesis will successfully translate in later phases of development (Carhart-Harris, 2023a; Greenway et al., 2020). If the oxymoronic notion of “non-psychedelic psychedelics” has substance, it may be via an action relevant to psychedelic “microdosing,” where very low doses of a psychedelic are taken semi-chronically (e.g., every other day) to achieve a desired psychological effect. It is conceivable that microdosing promotes PP. But evidence for the efficacy of psychedelic microdosing is presently mixed and inconclusive (Daldegan-Bueno et al., 2026; Szigeti et al., 2021; Kaertner et al., 2021). In comparison, the evidence for the safety and efficacy of high-dose psychedelic therapy for mental health improvement is weighty and compelling (Rieser et al., 2025; Carhart-Harris et al., 2021; Raison et al., 2023).

## 13 Summary, conclusions and future directions

In summary, we asked whether customary uses of the term “plasticity” within neuroscientific and mental health research have strayed, problematically, away from the term’s original dictionary definition as *changeability*. As with other topics, such as the integrity of “experimental blinding” (Szigeti et al., 2023) and the putative role of “expectancy” effects in driving positive responses to psychedelics (Szigeti et al., 2024), the particularities of psychedelic science and medicine are illuminating problematic theoretical assumptions and methodological conventions that have historically been ignored, neglected, or trivialized(Carhart-Harris et al., 2026). Relatedly, we argue that the meaning of “plasticity”—in the context of neuroscience and mental health research—requires a rethink.

Translating psychedelic science into routine care will require attention to implementation constraints. Psychedelic therapy is resource intensive: legal and regulatory access, therapist training and credentialing, screening, monitoring, integration, follow-up, adverse-event pathways, reimbursement, and public-health financing all shape whether these treatments can be delivered safely and equitably outside specialized research settings (Greenway et al., 2020; Belouin et al., 2022; Marseille et al., 2022). Thus, future tests of MR-P should examine not only whether a pro-plastic state is induced, but whether the surrounding care model is scalable, governable, affordable, and accessible. Such scaling will naturally introduce cost-effectiveness pressures. A streamlined (budget) model could aid scaling but compromise safety and efficacy. It is critically important that any such cost-cutting not render the model insufficiently safe.

As scientific and general audience publications on psychedelics, liberal policy changes, and use-prevalence (Yockey et al., 2020) continue to expand, it is important to address and resolve problematic conventions, lest they be the source of mistakes. In the worst of scenarios, paradigmatic confusion regarding the action of psychedelics could cause serious harm, as has happened in the past (Lee and Shlain, 1992). Regardless of whether ESBA proves to be the valuable biomarker of M-plasticity that we suspect it to be, attending to our understanding and use of “plasticity” in neuroscience and mental health research feels timely, necessary, and important.

## Data Availability

Underlying participant-level data are not publicly available at this stage because the relevant parent-study analyses and publications are not yet complete. Requests may be directed to the corresponding author and will be considered subject to participant consent, institutional ethics approval, and parent-study governance constraints.

## Glossary

ADP: activity-dependent plasticity
CRediT: Contributor Roles Taxonomy
DMT: N,N-dimethyltryptamine
DV: dependent variable
EBH: entropic brain hypothesis
EEG: electroencephalography
ESBA: entropy of spontaneous brain activity
fMRI: functional magnetic resonance imaging
LSD: lysergic acid diethylamide
LTD: long-term depression
LTP: long-term potentiation
LZc: Lempel-Ziv complexity
MEG: magnetoencephalography
MR-P: mediational and recalibrative plasticity
PCP: paradoxically canalizing plasticity
PP: plasticity proper
R-plasticity: recalibrative plasticity
SSRI: selective serotonin reuptake inhibitor
STDP: spike-timing-dependent plasticity
THC: tetrahydrocannabinol
VTA: ventral tegmental area
