# Supplementary material for: On the meaning of “plasticity” in neuroscience and mental health research and its relation to the action of psychedelic therapy
Source: Front Neurosci. 2026 Sep 1;20:1875339. doi: 10.3389/fnins.2026.1875339 (PMC13574699; doi:10.3389/fnins.2026.1875339)
Supplement: Supplementary file 1 [file Table_1.DOCX]

**Supplementary Table 1.** Working glossary and taxonomy of plasticity-related terms used or implied in the manuscript.

**Clean model:** Plasticity proper means changeability; neuroplasticity is neural change; M-plasticity opens a context-sensitive window; context helps determine whether that window closes into canalization or resolves through recalibration; MR-P names the constructive pathway when mediational changeability is linked to recalibrative movement toward plasticity proper.

| **Term** | **Working definition** |
| --- | --- |
| **Core Conceptual Terms** | |
| **Plasticity proper (PP)** | Plasticity in its basic dictionary sense: changeability, malleability, or the capacity of a phenotype, state, or system to be shaped or molded. In the manuscript, PP is the root concept being recovered and distinguished from neuroplasticity; a measured brain change does not automatically count as PP unless it implies or supports downstream changeability. |
| **Neuroplasticity** | An induced change in brain structure, function, activity, connectivity, synaptic organization, or neural dynamics. Neuroplasticity may participate in PP, but it is not equivalent to PP because neural change can support adaptive learning, maladaptive learning, canalization, or no demonstrated psychological changeability. |
| **Phenotypic plasticity** | The capacity of an organismal phenotype to vary in response to genetic, developmental, environmental, or contextual conditions. It provides a biological bridge between dictionary plasticity and psychological plasticity while preserving the core idea of changeability. |
| **Psychological plasticity** | Changeability of psychological states, traits, meanings, habits, beliefs, affective patterns, behavioral tendencies, or modes of self-world relation. This is the mental-health-relevant expression of PP, but its endpoint should be specified, such as flexibility, insight, emotional responsivity, behavior change, or well-being. |
| **M-plasticity** | Mediational plasticity: an acute state, process, or marker that increases changeability, lability, or context-sensitivity. M-plasticity opens a plasticity window and creates an opportunity for change, but it does not determine whether the eventual outcome will be adaptive, maladaptive, or durable. |
| **R-plasticity** | Recalibrative plasticity: downstream movement away from canalization and toward renewed flexibility, balance, integration, or adaptive responsiveness. R-plasticity describes where the plasticity window leads, and it is the component that aligns MR-P with plasticity proper. |
| **MR-P** | Mediational and recalibrative plasticity: the compound process in which an upstream mediational increase in changeability is linked to downstream recalibration. The term should be used when both the opening of a plasticity window and a movement toward renewed flexibility or balance are being claimed. |
| **PCP** | Paradoxically canalizing plasticity: neuroplastic, learning, or adaptation processes that culminate in reduced phenotypic changeability. PCP names the manuscript's central paradox: mechanisms popularly described as plasticity can, in some contexts, consolidate rigid or maladaptive phenotypes. |
| **Canalization** | Reduction of phenotypic variability into a more constrained, stable, or resistant pathway. In the manuscript, canalization is the conceptual inverse of PP, especially when learning, stress, reinforcement, or atrophy narrow the organism's future capacity for adaptive change. |
| **Chreode** | Waddington's term for a necessary or canalized developmental path within the epigenetic landscape. In this manuscript it functions as historical and conceptual background for canalization and phenotypic trajectory language, not as a new empirical claim. |
| **Allostatic recalibration** | A shift in regulatory set points, system organization, or adaptive balance through change. It is a conceptual neighbor of R-plasticity and annealing-like recalibration, but requires nuance because repeated or chronic allostatic demand can become allostatic load and contribute to pathology. |
| **Neural and Synaptic Terms** | |
| **Long-term potentiation (LTP)** | Enduring strengthening of synaptic transmission following repeated or coordinated activity. LTP is a classic marker of Hebbian neuroplasticity and acquisition learning; it can support adaptive learning but can also consolidate maladaptive associations. |
| **Long-term depression (LTD)** | Enduring weakening of synaptic transmission. LTD is complementary to LTP and can be relevant to learning, unlearning, and extinction depending on context. |
| **Hebbian plasticity** | Input-specific strengthening or weakening of neural associations through coordinated activation. Hebbian mechanisms are central to associative learning and become especially relevant to PCP when repeated activation consolidates maladaptive associations. |
| **Homosynaptic plasticity** | Plasticity at the specifically activated synapse or input pathway. In the manuscript, homosynaptic strengthening is easier to reconcile with PCP when it reinforces maladaptive associations or narrows the organism's future response repertoire. |
| **Heterosynaptic plasticity** | Plasticity affecting synapses or pathways beyond the specifically activated input. It may be more compatible with distributed recalibration than homosynaptic consolidation, although compatibility with PP depends on the empirical case and downstream phenotype. |
| **Metaplasticity** | Plasticity of plasticity: changes in the conditions, thresholds, or probability of later plastic change. It can bridge critical periods, psychedelic effects, and context sensitivity, but is broad and should be defined locally when used. |
| **Homeostatic plasticity** | Plasticity that stabilizes neural activity around functional ranges. It can counterbalance runaway potentiation or maladaptive reinforcement, but adaptive neural stabilization is not automatically evidence of psychological PP. |
| **Synaptic scaling** | A global or cell-wide adjustment of synaptic strengths to maintain stable activity. It is a homeostatic mechanism and should not be conflated with input-specific Hebbian strengthening. |
| **Spinogenesis** | Formation, growth, or remodeling of dendritic spines. Spinogenesis is a common anatomical marker in psychedelic and psychoplastogen literature, but spine growth does not by itself prove synaptogenesis, psychological plasticity, or mental-health benefit. |
| **Synaptogenesis** | Formation of synapses or synaptic contacts. It is related to spinogenesis but not identical with it; the term should be used when the cited assay supports synapse formation or synaptic function/contact. |
| **Dendritogenesis** | Growth or remodeling of dendritic structure. Like spinogenesis, dendritogenesis is visually persuasive as anatomical neuroplasticity, but structural change alone is not automatically evidence of enhanced PP. |
| **Synaptic pruning** | Elimination of synapses during development, learning, aging, or pathology. Pruning can contribute to refinement, but excessive or stress-related pruning may contribute to narrowing, atrophy, or canalization depending on context and downstream function. |
| **Psychedelic, ESBA, and Clinical Terms** | |
| **Psychedelic therapy** | Administration of a psychedelic medicine within structured psychological support, preparation, procedural context, and integration. In the manuscript, therapy should not be reduced to drug effect alone because context is central to whether M-plasticity culminates in MR-P or PCP. |
| **Psychoplastogen** | Term used for compounds that rapidly promote structural or functional neural plasticity, often cortical spinogenesis. The manuscript critiques the term when evidence is only neuroanatomical, because 'psycho-' implies psychological plasticity that may not have been demonstrated. |
| **Neuroplastogen** | A compound defined by its ability to induce neuroplastic changes. This is more precise than psychoplastogen when the evidence concerns neural structure or function rather than psychological plasticity or plasticity proper. |
| **Cortical spinogenesis** | Formation or growth of dendritic spines in cortical tissue. It may suggest a substrate for recalibration, especially when contrasted with homosynaptic canalization, but it does not by itself prove enhanced psychological changeability or mental-health improvement. |
| **Critical-period reopening** | Transient restoration of developmental-like windows of heightened sensitivity to experience. This is a complementary model for psychedelic action and context dependence, but human homology remains an interpretive step when evidence is preclinical. |
| **Sensitive period** | A developmental window during which experience has especially strong effects, but with less rigid timing than a critical period. The term can soften critical-period language while preserving the idea of heightened contextual input effects. |
| **Post-stroke plasticity window** | A time-limited period after stroke during which rehabilitation input can have heightened effects. It provides an adult analogy for bounded, context-dependent plasticity windows, but should remain an analogy unless specific psychedelic stroke evidence is being cited. |
| **ESBA** | Entropy of spontaneous brain activity: entropy or complexity of spontaneous EEG/MEG activity, often recorded during eyes-closed rest. ESBA is framed as a candidate acute-state marker of psychedelic action and M-plasticity, and should be treated as a marker or predictor unless causal evidence is direct. |
| **Lempel-Ziv complexity (LZc)** | A compression-based algorithm commonly used to estimate signal complexity or entropy in EEG/MEG time series. In the manuscript, LZc is an operational index used in ESBA work rather than the whole construct. |
| **Entropic brain hypothesis (EBH)** | A model proposing that brain entropy has meaningful relationships with conscious-state richness or expansiveness. EBH provides a theoretical bridge between ESBA and phenomenology, but should not substitute for clinical outcome evidence. |
| **Phenomenal consciousness** | The felt field, size, richness, or expansiveness of subjective experience. In the manuscript, ESBA may index aspects of phenomenal consciousness, but this claim should remain distinct from therapeutic outcome claims. |
| **Psychological insight** | Actionable or meaningful understanding arising during or after the psychedelic state. It is a candidate mediator between acute ESBA and later well-being, and should be distinguished from emotional release, mystical-type experience, and outcome itself. |
| **Emotional release** | Acute affective discharge or processing during therapy. Emotional release can predict or mediate therapeutic outcomes but is not identical with insight, mystical-type experience, or long-term psychological change. |
| **Mystical-type / peak experience** | Highly meaningful, expansive, transcendent, or personally significant acute subjective experience. It is an important phenomenological marker in psychedelic therapy literature, but its necessity and specificity remain debated. |
| **Context-dependent medicine** | A medicine whose direction, magnitude, variability, durability, or valence of effect depends strongly on psychological, social, procedural, or environmental context. This phrase captures the MR-P framing of psychedelics as context-sensitive rather than automatically therapeutic. |
| **Pathways and Process Terms** | |
| **Neuroplasticity without PP** | A case in which brain structure or function changes, but downstream phenotypic or psychological changeability is not demonstrated. A cortical spine change without evidence of psychological changeability would remain neural change only. |
| **M-plasticity without R-plasticity** | A case in which an acute changeability window opens but does not culminate in recalibration. Insight, sensitivity, or lability may occur without durable constructive change. |
| **Plasticity-mediated canalization** | A pathway in which changeability is recruited into repeated, reinforced, or constrained patterns, producing reduced flexibility. This is the process-level logic behind PCP. |
| **Annealing-like recalibration** | A pathway in which changeability occurs in supportive conditions and permits reintegration, rebalancing, or renewed flexibility. This is the constructive endpoint that makes MR-P consistent with PP. |
| **Context sensitivity** | The degree to which surrounding conditions shape the direction, magnitude, variability, durability, or valence of outcome. It is a key prediction of M-plasticity and a central reason psychedelics are treated as context-dependent medicines. |
| **Differential susceptibility** | Trait-like variation in sensitivity to environmental influence. It is relevant to inherent plasticity and may help explain why the same M-plasticity-promoting intervention produces different outcomes across individuals. |
| **Environmental sensitivity** | Responsiveness to contextual or environmental input. Environmental sensitivity can function as a baseline moderator of M-plasticity outcomes. |
| **Inherent plasticity / trait plasticity** | Baseline individual differences in the capacity to enter, tolerate, or benefit from plasticity-promoting states. In the metallurgical analogy, this maps loosely onto ductility versus brittleness before heat is applied. |
| **Metallurgical Analogy Terms** | |
| **Heat** | In the analogy, heat is the intervention-induced state that increases workability or changeability. It maps to M-plasticity, not to literal temperature. |
| **Workability** | The practical capacity of heated material to be shaped. It maps to the acute plasticity window in which context can guide change. |
| **Ductility** | The capacity to deform without breaking. It maps to inherent or trait plasticity, especially the capacity to tolerate or benefit from plasticity induction. |
| **Brittleness** | The tendency to fracture, destabilize, or decompensate under force rather than deform constructively. It maps to elevated psychological vulnerability or risk under M-plasticity. |
| **Controlled work / force** | Shaping applied under suitable conditions. It maps to supportive ecology, therapeutic container, task practice, integration, and other forms of contextual guidance. |
| **Haphazard force** | Poorly structured or adverse shaping during a period of heightened workability. It maps to contexts that may drive PCP or maladaptive outcomes. |
| **Residual stress** | Internal stress retained from prior deformation or processing. It is a possible analogy for unresolved stress, prior adverse shaping, or canalized tension, but should be used cautiously. |
| **Grain structure** | Internal material organization that influences properties. It can loosely analogize underlying system organization, but should not be overextended. |
| **Annealing** | A metallurgical process in which heating and controlled cooling relieve internal stress and restore useful properties such as ductility or workability. In the manuscript, it maps to recalibrative, PP-aligned recovery of flexibility. |
| **Morphological change** | Change in form after heat plus force or context. In the model, this maps to phenotypic change, which may be adaptive or maladaptive depending on context and endpoint. |
| **Outcome and Measurement Terms** | |
| **Psychological change** | State or trait change in insight, flexibility, meaning, emotion, sensitivity, equanimity, or self-world relation. It is closest to the manuscript's psychological plasticity construct. |
| **Mental-health change** | Change in well-being, symptoms, distress, functioning, impairment, or clinical status. It is often the clinical outcome of interest but should not be inferred from neural plasticity alone. |
| **Behavioral change** | Observable change in action, habits, avoidance, substance use, rehabilitation practice, social behavior, or functioning. It may be downstream of psychological change but should not be assumed. |
| **Phenotypic change** | Broad organism-level change spanning psychological, behavioral, biological, and clinical features. The term is useful as an umbrella but should be specified where possible. |
| **Psychological flexibility** | The capacity to adapt thoughts, feelings, and actions in response to values and context. It is a candidate psychological PP outcome. |
| **Openness** | Trait openness to experience. It is a candidate downstream psychological plasticity marker, especially in psychedelic research. |
| **Absorption** | Capacity for deep attentional or experiential immersion. It may be a moderator or marker relevant to psychedelic response. |
| **Terms to Use Carefully** | |
| **Plasticity** | Use carefully because it can obscure whether the claim concerns change, changeability, neuroplasticity, psychological plasticity, or improved mental health. Specify PP, neuroplasticity, M-plasticity, R-plasticity, MR-P, or PCP when possible. |
| **Maladaptive plasticity** | A useful legacy shorthand, but conceptually imprecise under the manuscript's strict PP definition. Consider 'plasticity-mediated canalization' or 'PCP' when the endpoint is reduced changeability. |
| **Reset** | Potentially misleading because it can imply a return to a fixed prior set point. Pair with recalibration, renewed flexibility, or allostatic adjustment if that is the intended meaning. |
| **Biomarker of plasticity** | Potentially overbroad because neural change does not automatically imply psychological or phenotypic changeability. Specify whether the marker concerns neuroplasticity, M-plasticity, MR-P, or PP. |
| **Synaptogenesis / spinogenesis** | Related but not interchangeable. Use the term supported by the cited assay, and avoid treating spine growth as proof of synapse formation or psychological plasticity unless the evidence supports it. |
| **Positive mental-health change** | A broad outcome class. Where possible, distinguish psychological, clinical, behavioral, and phenotypic endpoints. |
